# Transient paraproteinemia after allogeneic hematopoietic stem cell transplantation is an underexplored phenomenon associated with graft versus host disease

**DOI:** 10.18632/oncotarget.22462

**Published:** 2017-11-15

**Authors:** Corinne C. Widmer, Stefan Balabanov, Urs Schanz, Alexandre P.A. Theocharides

**Affiliations:** ^1^ Division of Hematology, University Hospital Zurich and University of Zurich, Zurich, Switzerland

**Keywords:** paraprotein, allo-HSCT, GvHD, myeloma

## Abstract

The clinical and biological relevance of a paraprotein that newly arises after allogeneic hematopoietic stem cell transplantation (allo-HSCT) in non-myeloma patients is unknown. In this study, the incidence, the course, and the clinical impact of paraproteins found after allo-HSCT were investigated in a cohort of 383 non-myeloma patients. Paraproteinemia after allo-HSCT was more frequent (52/383 patients, 14%) than the reported incidence of monoclonal gammopathy of unknown significance (MGUS) in age-matched healthy subjects and, in contrast to MGUS, did not correlate with age. In most patients (32/52, 62%), the paraprotein appeared transiently within the first year after allo-HSCT with a median duration of 6.0 months. Post-allo-HSCT paraproteinemia was significantly associated with graft versus host disease (GvHD) and correlated with a survival benefit within the first year, but not after five years following allo-HSCT. Importantly, patients with post-allo-HSCT paraproteinemia did not progress into a plasma cell myeloma as observed for MGUS inferring a distinct pathogenic mechanism. Skewing of lymphocyte subpopulations and alterations in cytokine levels in GvHD may explain the expansion of a specific plasma cell subset in non-myeloma patients undergoing allo-HSCT. Our data suggests that paraproteinemia after allo-HSCT is a reactive phenomenon rather than the consequence of clonal plasma cell transformation.

## INTRODUCTION

Paraproteins are abnormal immunoglobulins detected by immunofixation and protein electrophoresis. A monoclonal gammopathy of unknown significance (MGUS) is characterized by the production of a paraprotein by clonal, genetically aberrant plasma cells [1]. While the detection of a paraprotein per se is abnormal, its clinical relevance depends on the plasma cell mass and end organ damage. In healthy subjects, the incidence of paraproteinemia increases with age and confers an increased risk for the development of plasma cell myeloma [2]. IgG is the most frequent paraprotein subtype while the incidence of IgM and IgA paraproteins is significantly lower, but associated with a higher risk of transformation to plasma cell myeloma [3].

The appearance of paraproteins following allogeneic hematopoietic stem cell (allo-HSCT) and solid organ transplantation has been reported previously [4–9]. However, the clinical and prognostic relevance of post-transplantation paraproteinemia remains unclear. Factors reported to be associated with the development of paraproteinemia following allo-HSCT include an alemtuzumab-based conditioning regimen, reactivation of cytomegalovirus (CMV), and occurrence of chronic graft versus host disease (GvHD) [6, 7].

In this large retrospective study, we investigated the development of post-allo-HSCT paraproteinemia in 383 non-myeloma patients treated with allo-HSCT and aimed at identifying predictive factors and clinical parameters associated with this phenomenon.

## RESULTS

**Figure 1:**
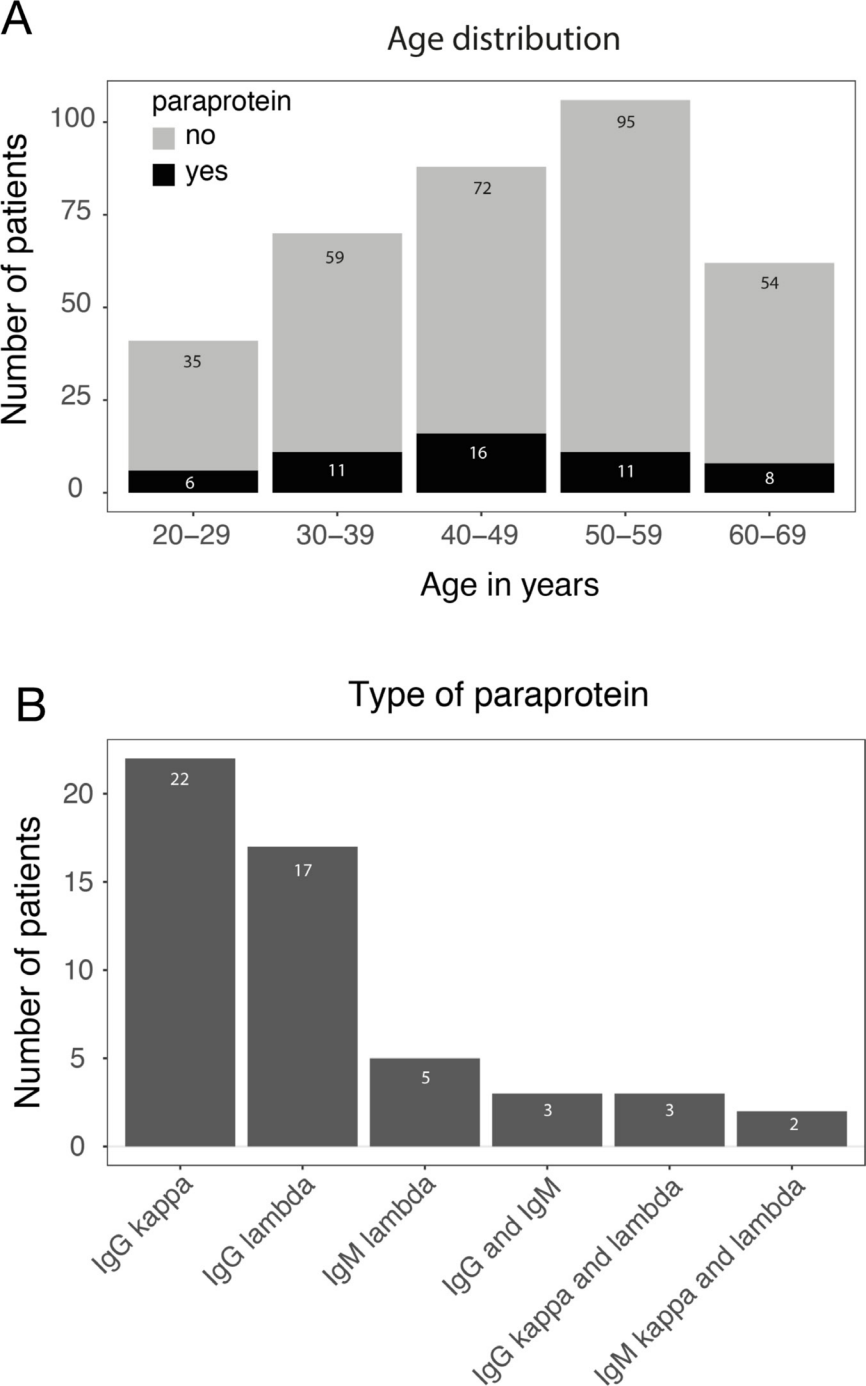
Characterization of post-allo-HSCT paraproteinemia (**A**) Number of patients with (black) and without (grey) paraproteins after allo-HSCT stratified according to the age range. (**B**) Post-allo-HSCT paraprotein subtypes.

**Figure 2:**
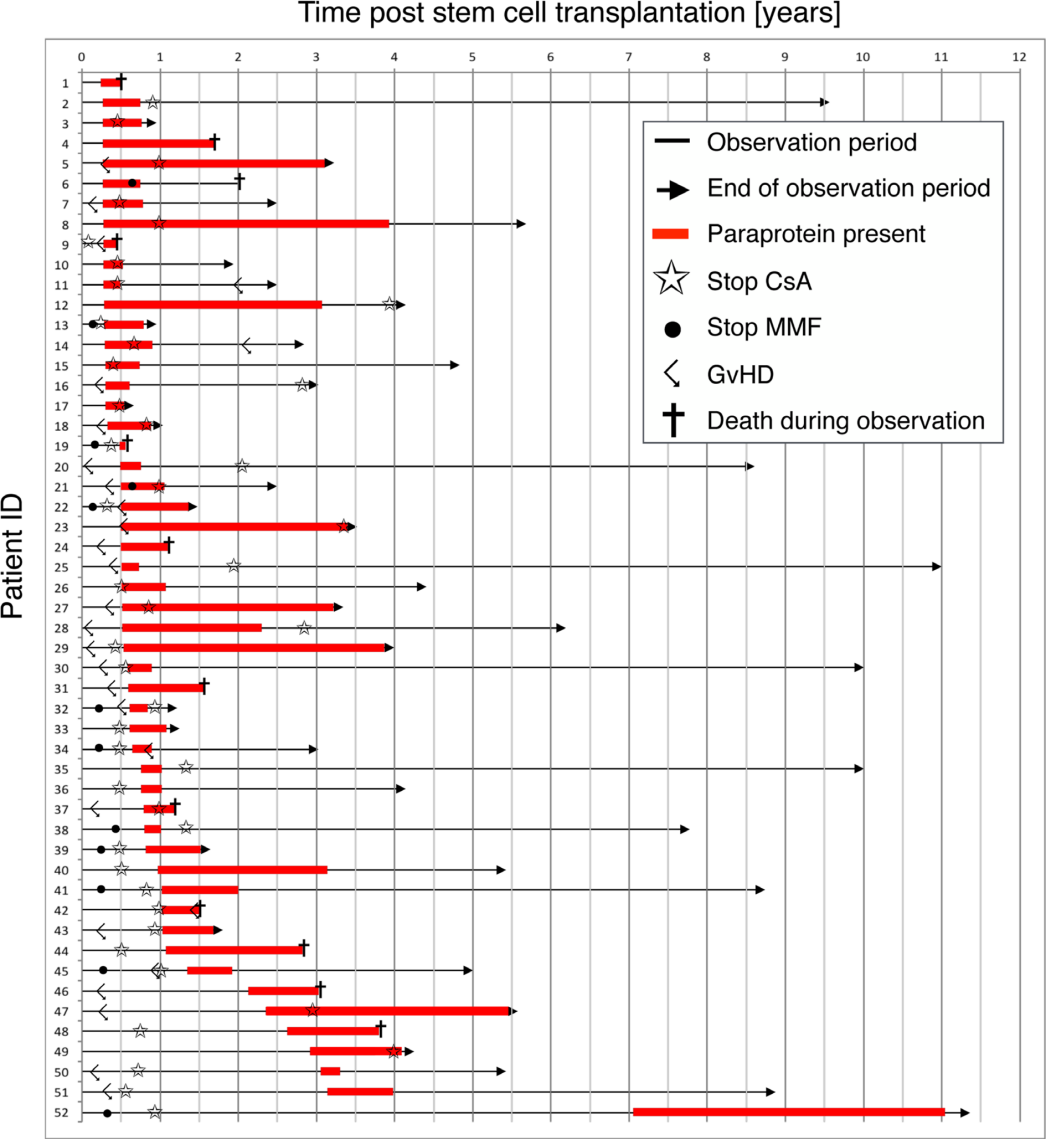
Development of paraproteinemia over time Each arrow represents the observation period over time of an individual patient with paraproteinemia in the study. The red bar indicates the presence of a paraprotein. Star, Cessation of ciclosporin A; Dot, Cessation of mycophenolate mofetil; Angled arrow, diagnosis of GvHD; Cross, death during observation period.

We analyzed a cohort of 383 non-myeloma patients who underwent allo-HSCT for the development of paraproteinemia after HSCT. Paraproteinemia was detected in 52/383 (14%) patients and was equally distributed among age groups (*p =* 0.723) (Figure 1A). Most paraproteins were of IgG subtype (39/52, 75%) with an equal distribution between kappa and lambda light chain expression (Figure 1B). An IgM paraprotein was found in 5/52 (9.6%) patients. In 8/52 (15.4%) cases, two paraproteins were detected in the same patient (IgG kappa/IgG lambda, IgM kappa/IgM lambda and IgG/IgM). However, no significant difference in clinical parameters (see below) and survival was observed between patients with one or two paraproteins (data not shown). Almost all paraproteins (50/52, 96%) detected by immunofixation were not quantifiable by protein electrophoresis due to the low level of paraprotein expression, and bone marrow histology did not reveal infiltration by plasma cell myeloma or lymphoma. In the majority of cases (40/52, 77%), paraproteinemia appeared within the first year after allo-HSCT and the median time to detection of a paraprotein was 6.0 months post-allo-HSCT (range: 0.24–7.05 years, Figure 2). The majority of paraproteins (32/52, 62%) were transient with a median period of detection of 6.0 months (range 2.5–46.8 months). However, in 20/52 (38%) patients, the paraproteinemia persisted until death (*n =* 11) or the end of the observation period (*n =* 9). The median observation period of all patients with paraprotein was 37 months (range 5.4–137.1 months). The most frequent cause of death was relapse of the underlying disease (11 patients), while one patient died from GvHD. No evolution to plasma cell myeloma or B-cell lymphoma was observed in patients with post-allo-HSCT paraproteinemia. Accordingly, laboratory parameters associated with the development of plasma cell myeloma did not deteriorate between the first and the last detection of paraproteinemia (Supplementary Table 1 and Supplementary Figure 1).

**Figure 3:**
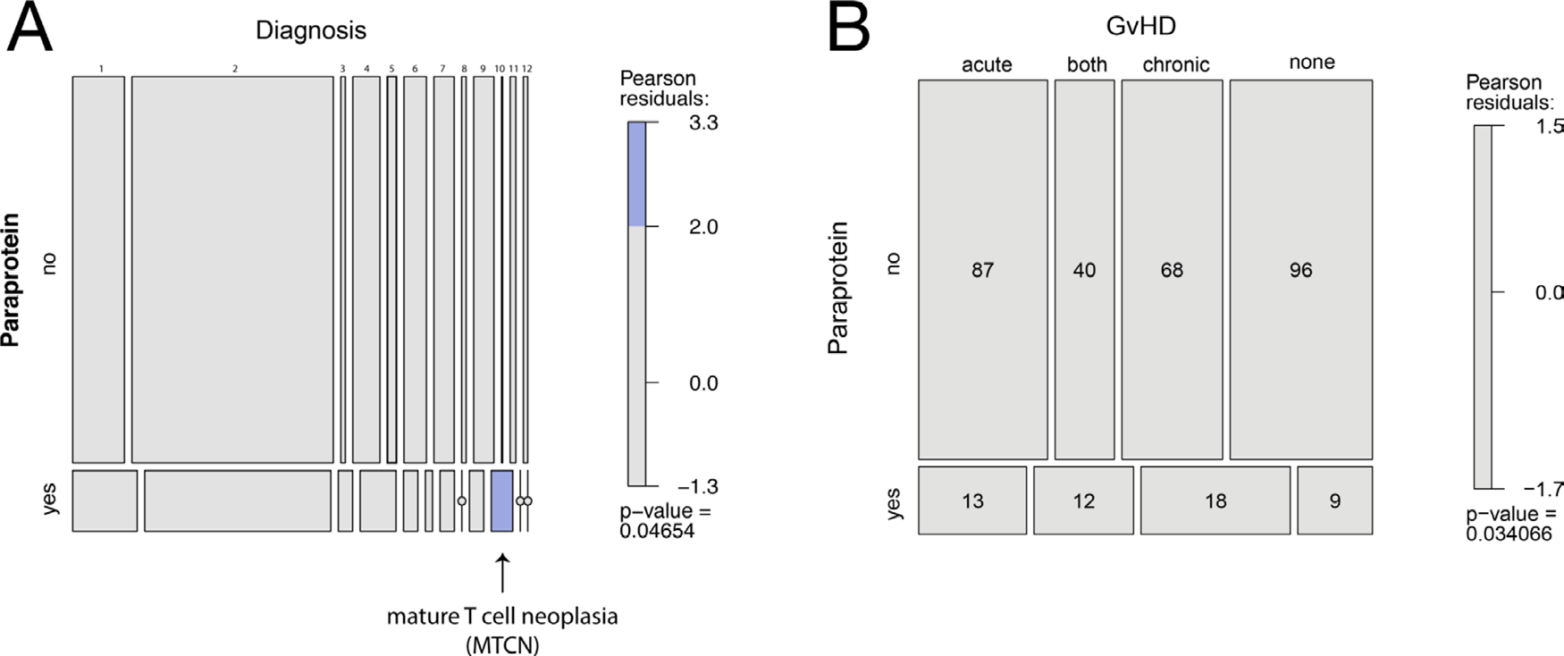
Association of post-allo-HSCT paraproteinemia and clinical parameters depicted in mosaic plots (**A**) Association of post-allo-HSCT paraproteinemia and diagnoses according to Table 1. The mosaic plots graphically display data on a log-linear scale. Each horizontal box from left to right represents a diagnosis as presented in Table 1: 1) ALL, acute lymphoblastic leukemia, 2) AML, acute myeloid leukemia, 3) CLL, chronic lymphocytic leukemia, 4) CML, chronic myeloid leukemia, 5) HL, Hodgkin lymphoma, 6) MBCN, mature B-cell neoplasms, 7) MDS, myelodysplastic syndrome, 8) MDS/MPN, myelodysplastic/myeloproliferative neoplasms, 9) MPN, myeloproliferative neoplasms, 10) MTCN, mature T-cell neoplasms including Sézary syndrome and mycosis fungoides, 11) PID, Primary immunodeficiency, 12) SAA, severe aplastic anemia. The blue box indicates a significant association for patients with mature T cell neoplasms (MTCN) and post-allo-HSCT paraproteinemia. The small open dots indicate that no patient was identified in the subgroup. (**B**) Association of post-allo-HSCT paraproteinemia and GvHD.

To identify factors that may predict or be causative for paraprotein development following allo-HSCT we then correlated clinical parameters with the appearance of post-allo-HSCT paraproteinemia. The majority of patients in the study were treated for acute leukemia, but no association was identified between the underlying disease and the appearance of paraproteins (260/383, 68%, Table 1).However, using a Chi-squared test and a log-linear model a significant association was identified between the detection of a paraprotein and a small group of patients with mature T-cell lymphoma, but not with other diseases (Table 1 and Figure 3A).

**Table 1:** Characteristics of patients with post-allo-HSCT paraproteinemia

Parameter	No paraprotein detected	Paraprotein detected	*p*-value
**Age (y)**			
**Median**	48	45	0.723
**IQR**	36.0–57.5	39.8–53.0	
**Gender (*n*, %)**			
Female	143 (43.2)	27 (51.9)	0.299
Male	188 (56.8)	25 (48.1)	
**Diagnosis (*n*, %)**			
ALL	46 (13.9)	9 (17.3)	0.047
AML	179 (54.1)	26 (50.0)	
CLL	4 (1.2)	2 (3.8)	
CML	24 (7.3)	5 (9.6)	
HL	8 (2.4)	2 (3.8)	
MBCN	20 (6.0)	1 (1.9)	
MDS	18 (5.4)	2 (3.8)	
MDS/MPN	4 (1.2)	0 (0.0)	
MPN	18 (5.4)	2 (3.8)	
MTCN	1 (0.3)	3 (5.8)	
PID	5 (1.5)	0 (0.0)	
SAA	4 (1.2)	0 (0.0)	
**Conditioning (*n*, %)**			
MAC	81 (24.5)	13 (25.0)	0.225
MAC+ATG	74 (22.4)	16 (30.8)	
RIC	31 (9.4)	1 (1.9)	
RIC+ATG	145 (43.8)	22 (42.3)	
**Donor (*n*, %)**			
MRD	157 (47.4)	21 (40.4)	0.127
MUD	135 (40.8)	29 (55.8)	
Mismatched	29 (8.8)	1 (1.9)	
Haploidentical	10 (3.0)	1 (1.9)	
**Stem cell source (*n*, %)**			
Bone marrow	50 (15.1)	11 (21.2)	0.381
Peripheral blood	276 (83.4)	41 (78.8)	
Cord blood	5 (1.5)	0 (0.0)	
**GvHD (*n*, %)**			
Acute	87 (29.9)	13 (25.0)	0.034
Chronic	68 (23.4)	18 (34.6)	
Both	40 (13.7)	12 (23.1)	
None	96 (33.0)	9 (17.3)	
**CMV (*n*, %)**			
Reactivation	69 (23.3)	15 (28.8)	0.494
No reactivation	227 (76.7)	37 (71.2)	

IQR, interquartile range; Diagnoses: ALL, acute lymphoblastic leukemia; AML, acute myeloid leukemia; CLL, chronic lymphocytic leukemia; CML, chronic myeloid leukemia; HL, Hodgkin lymphoma; MBCN, mature B-cell neoplasms; MDS, myelodysplastic syndrome; MDS/MPN, myelodysplastic/myeloproliferative neoplasms; MPN, myeloproliferative neoplasms; MTCN, mature T-cell neoplasms including Sézary syndrome and mycosis fungoides; PID, Primary immunodeficiency; SAA, severe aplastic anemia. Conditioning regimens: MAC, myeloablative conditioning; MAC+ATG, myeloablative conditioning with anti-thymocyte globulin (Fresenius); RIC, reduced intensity conditioning; RIC+ATG, reduced intensity conditioning with anti-thymocyte globulin. Donor: MRD, mached related donor; MUD, matched unrelated donor (10/10); Mismatched, non-HLA-identical donor; Haploidentical, haploidentical family donor; GvHD, graft versus host disease; CMV, cytomegalovirus.

**Figure 4:**
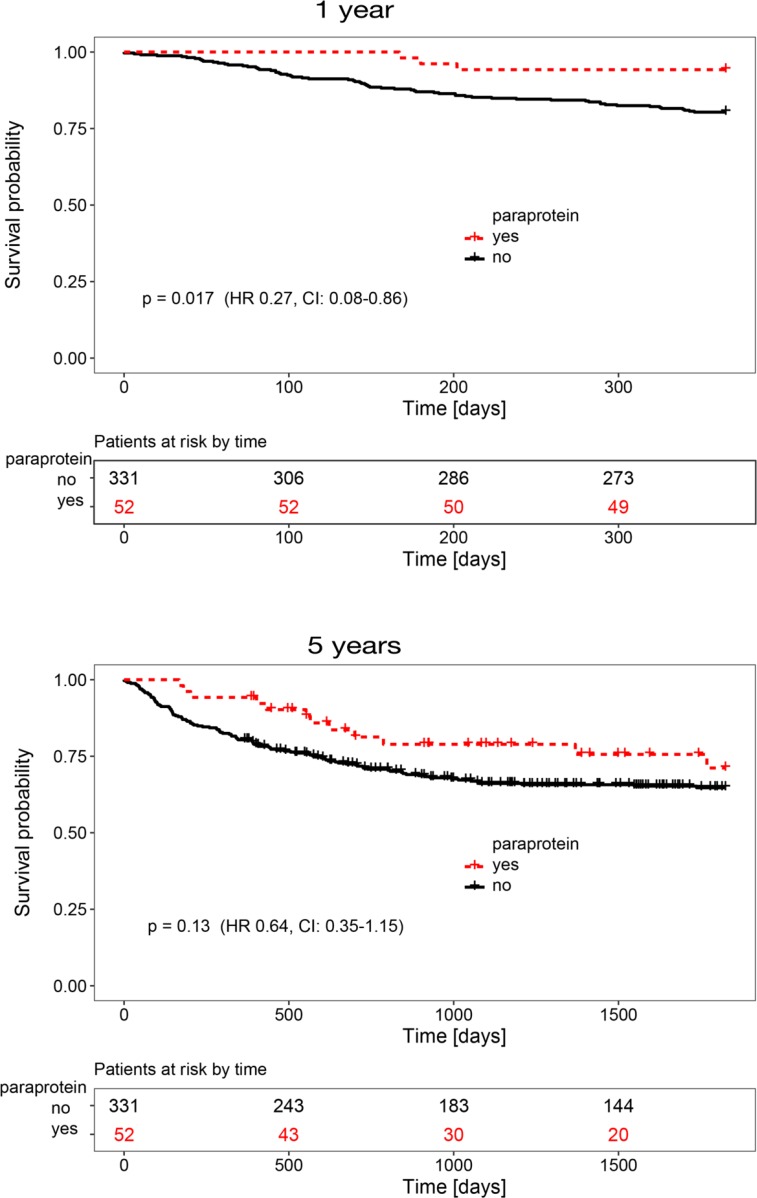
Kaplan-Meier survival analysis for patients with (dashed red line) or without (black line) post-allo-HSCT paraproteinemia (**A**) Cumulative survival one year after allo-HSCT. (**B**) Cumulative survival five years after allo-HSCT.

The median age of patients with paraproteinemia was 45 years (Table 1) and paraproteinemia was not associated with gender (*p =* 0.299). In 12 patients, paraproteinemia occurred after the first year following allo-HSCT (Figure 2), but we did not find significant differences in clinical parameters and survival between patients with early (i.e. <12 months post-allo-HSCT) or late (i.e. >12 months post-allo-HSCT) paraproteinemia (data not shown). An increased incidence of post-allo-HSCT paraproteinemia has been reported after alemtuzumab-based conditioning [6, 7]. In this study, only five patients obtained alemtuzumab as part of their conditioning regimen, but none of them developed paraproteinemia (Supplementary Table 2). Furthermore, the conditioning regimen and anti-thymocyte globulin (ATG) pretreatment were not associated with the development of paraproteinemia in the binary logistic regression analysis in our study (*p =* 0.225). In addition, neither the hematopoietic stem cell source (*p =* 0.381), nor the type of donor (*p =* 0.127) or the reactivation of CMV (*p =* 0.494) had an impact on the appearance of paraproteinemia. Since none of the patients with paraproteinemia developed a clonal plasma cell disorder, we hypothesized that post-allo-HSCT paraproteinemia was the consequence of a perturbed immune system in patients with GvHD. Indeed, the χ^2^ test for independency and the binary logistic regression both revealed an association between paraproteinemia and GvHD (*p =* 0.034 and *p =* 0.046, respectively, Table 1 and Figure 3B) supporting a causative role for GvHD in post-allo-HSCT paraproteinemia. Finally, the Kaplan-Meier survival analysis showed a statistically significant survival benefit in the first year after allo-HSCT for patients with paraproteinemia (*p =* 0.017, HR = 0.27, CI = 0.08–0.86), which was not observed five years following allo-HSCT (*p =* 0.13, HR = 0.64, CI = 0.35–1.15) (Figure 4).

## DISCUSSION

In this retrospective study, we investigated 383 non-myeloma patients treated with allo-HSCT and found a high incidence of post-allo-HSCT paraproteinemia compared to the incidence of MGUS in age-matched healthy subjects [3]. However, and in contrast to MGUS, none of the patients with paraproteinemia developed plasma cell myeloma or B-cell lymphoma during the observation period suggesting a distinct pathogenic mechanism. This hypothesis is also supported by the observation that 62% of post-allo-HSCT paraproteins were only detected transiently in our cohort. In our study 3/4 patients with mature T-cell lymphoma developed paraproteinemia following allo-HSCT, a finding that needs to be validated in a larger cohort of patients.

Interestingly, we found a significant association between paraprotein development and the occurrence of GvHD as observed previously in a smaller cohort [6]. Disruption of B-cell homeostasis has been implicated in the pathogenesis of GvHD [10–12]. Patients with chronic GvHD (cGvHD) produce excessive levels of B-cell activating factor (BAFF) which promotes B-cell survival and maturation and is associated with the production of pre-germinal center B-cells and post-germinal center plasma-like cells in patients with cGvHD [13]. Moreover, increased levels of BAFF are associated with increased autoantibody production [14]. Therefore, altered B-cell homeostasis following allo-HSCT may lead to aberrant production of immunoglobulins in GvHD as observed in our study. In addition, increased generation of a T-follicular helper cell subset that produces high levels of IL-17 and IL-21 and promotes B-cell immunoglobulin secretion may contribute to production of paraproteins [15]. Alterations in T-cell subpopulations may also underlie paraprotein generation in patients receiving allo-HSCT following an alemtuzumab-conditioning as reported previously [6, 7]. Post-allo-HSCT paraproteinemia was significantly associated with a survival benefit during the first year following allo-HSCT, but not at later time-points. Since most paraproteins appeared transiently during the first year after allo-HSCT and correlated with the occurrence of GvHD post-allo-HSCT paraproteinemia may hint towards the presence of a graft versus leukemia effect, which could explain the survival benefit observed in the investigated cohort.

In summary, our study investigates the largest non-myeloma patient cohort to date treated by allo-HSCT for the development of paraproteinemia and suggests an association with GvHD. Importantly, post-allo-HSCT paraproteinemia is mostly transient and does not translate into development of plasma cell myeloma or lymphoma. Future studies will determine the role of GvHD-associated paraproteinemia in the prediction of disease-free survival and investigate the involvement of specific lymphocyte subsets in the pathogenesis of this transient phenomenon.

## MATERIALS AND METHODS

### Patients

The study was approved by the local ethics committee (BASEC-Nr. 2016–01139). 383 patients who underwent allo-HSCT in the Division of Hematology at the University Hospital Zurich, Switzerland, between 2004 and 2014 were retrospectively investigated for the development of post-allo-HSCT paraproteinemia. The following patients were excluded from the study; patients with plasma cell myeloma, autoimmune disease, paraproteinemia before allo-HSCT and patients without documented negative immunofixation prior to allo-HSCT. The observation period started with the date of the allo-HSCT and ended December 31, 2015 or with the patient’s death. The patient characteristics are outlined in Table 1.

### Serum proteins electrophoresis measurement

Immunofixation and serum protein electrophoresis was performed before and regularly every 3 to 6 months after allo-HSCT. Proteins were separated on an agarose-gel followed by immunoprecipitation *in situ* with monospecific antisera against heavy and light chains (EasyFix Interlab G26, Apteq Switzerland). The gels were interpreted by two independent laboratory technicians. Paraproteins were quantified by densitometry. For the purpose of the study, only monoclonal paraproteins detected by immunofixation were used for correlative studies.

### Conditioning regimen and GvHD prophylaxis

The conditioning regimens used in the study are outlined in Supplementary Table 2. GvHD prophylaxis for all patients consisted of intravenous ciclosporin A (CsA) until engraftment of donor hematopoiesis, followed by oral application with a target whole blood concentration of 150–250 μg/l. Patients with myeloablative conditioning (MAC) received three to four doses of methotrexate (MTX, 15 mg/m^2^) on day 1 and 10 mg/m^2^ on day 3, 6 and 11 after allo-HSCT. In addition, mycophenolic mofetil (MMF, 2 g/d) was given to patients with reduced intensity conditioning (RIC) until day 28 in related donor and day 56 in unrelated donor allo-HSCT. In the absence of GvHD immunosuppression was tapered starting at day 100 following allo-HSCT.

### Graft versus host disease

Acute (aGvHD) and chronic GvHD (cGvHD) were defined according to the National Institutes of Health Consensus Development Project criteria [16]. GvHD of the eye and skin was diagnosed clinically. GvHD of the intestine and the liver was documented by histological analysis. Documentation on GvHD development was not available in 40 patients.

### Cytomegalovirus reactivation

CMV copy number quantification was performed twice weekly by polymerase chain reaction for the first 120 days following allo-HSCT. CMV reactivation was defined as a CMV copy number increase above 1000/µl. Patients with documented CMV reactivation obtained antiviral treatment. Documentation on CMV reactivation was not available in 35 patients.

### Statistical analysis

All statistical analyses were performed using the statistical software environment R v.3.2.2 (www.r-project.org) with the R packages tableone 0.8.1, compareGroups, vcd, survival and survminer. Comparison of categorical data between groups was performed with the Chi-squared test. Furthermore, categorical data for paraprotein, diagnosis and GvHD were analyzed using log-linear modeling and were visualized by mosaic plots. For the estimation of survival curves Kaplan-Meier methods were used. Comparison of overall survival between groups of patients with or without paraproteinemia was performed using the log-rank test and Cox’s proportional hazard model (Cox regression).

## SUPPLEMENTARY MATERIALS FIGURE AND TABLES



## Abbreviations

ALL: Acute lymphoblastic leukemia
Allo-HSCT: Allogeneic hematopoietic stem cell transplantation
AML: Acute myeloid leukemia
ATG: Anti-thymocyte globulin
CLL: Chronic lymphocytic leukemia
CML: Chronic myeloid leukemia
CMV: Cytomegalovirus
CsA: Ciclosporin A
GvHD: Graft versus host disease
HL: Hodgkin lymphoma
IQR: Interquartile range
MAC: Myeloablative conditioning
MBCN: Mature B-cell neoplasms
MDS: Myelodysplastic syndrome
MDS/MPN: Myelodysplastic/myeloproliferative neoplasms
MGUS: Monoclonal gammopathy of unknown significance
MMF: Mycophenolic mofetil
MPN: Myeloproliferative neoplasms
MRD: Mached related donor
MTCN: Mature T-cell neoplasms including Sézary syndrome and mycosis fungoides
MTX: Methotrexate
MUD: matched unrelated donor (10/10)
PID: Primary immunodeficiency
RIC: Reduced intensity conditioning
SAA: Severe aplastic anemia
