# Supplementary material for: Transient paraproteinemia after allogeneic hematopoietic stem cell transplantation is an underexplored phenomenon associated with graft versus host disease
Source: Oncotarget. 2017 Nov 15;8(63):106333–41. doi: 10.18632/oncotarget.22462 (PMC5739737; doi:10.18632/oncotarget.22462)
Supplement: Supplementary file 1 [file oncotarget-08-106333-s001.pdf]

Transient paraproteinemia after allogeneic hematopoietic stem cell transplantation is an underexplored phenomenon associated with graft versus host disease

SUPPLEMENTARY MATERIALS

Supplementary Table 1: Parameters associated with the progression to plasma cell myeloma compared between the first and the last time point of paraprotein detection

Parameter	Normal range	First paraprotein	IQR	Last paraprotein	IQR	*p*-value
Creatinine (mg/l)	0.50–1.12	1.14	0.94–1.29	1.10	0.91–1.24	0.376
eGFR (ml/min)	>60	70	58–81	69	62–84	0.364
Albumin (g/l)	40–49	43	40–44	43	41–45	0.516
Calcium (mmol/l)	2.09–2.54	2.36	2.32–2.45	2.39	2.30–2.45	0.880
Hemoglobin (g/l)	117–170	116	107–124	123	113–133	0.033

The median and the interquartile range (IQR) for each parameter are indicated. eGFR, estimated glomerular filtration rate.

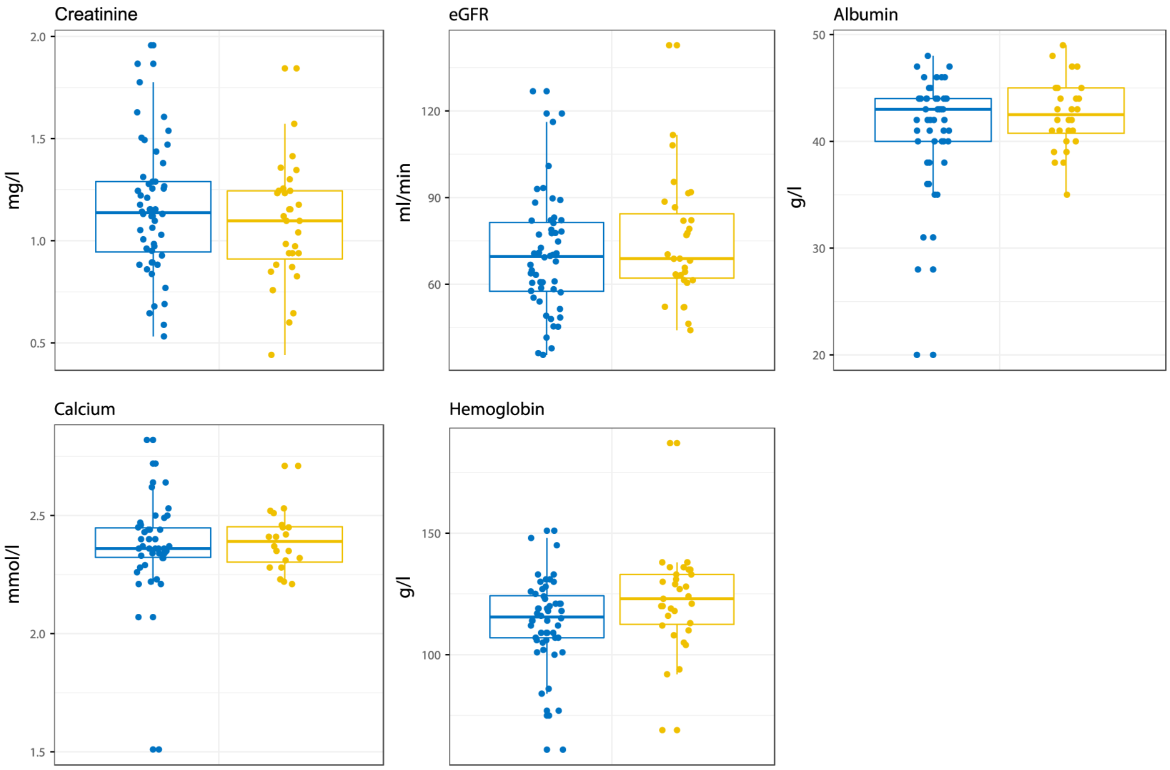

Supplementary Figure 1: Parameters associated with the progression to plasma cell myeloma compared between the first (blue) and the last (yellow) time point of paraprotein detection. eGFR, estimated glomerular filtration rate.

Supplementary Table 2: The table shows the correlation between the conditioning regimens used in the study and the presence of post-allo-HSCT paraproteins

Conditioning intensity	Conditioning regimen	Total patients	Paraprotein detected (%)
**Myeloablative**	Bu-Cy	32	6 (19)
	Bu-Cy-ATG	21	3 (14)
	Bu-Cy-Bu	2	0
	Cy-ATG-TBI short	37	8 (22)
	Cy-ATZ-TBI	1	0
	Cy-TBI short	47	5 (11)
	Cy-TBI-long	12	2 (17)
	Flu-Bu	1	1 (100)
	Flu-Bu-ATG	7	0
	Flu-Bu-ATG (haplo)	3	1 (33)
	Flu-Mel-ATG	11	0
	VP16-Cy-ATG-TBI	6	2 (33)
	VP16-Cy-TBI	9	1 (1)
**Reduced intensity**	FLAMSA	3	0
	Flu-Bu	7	0
	Flu-Bu-ATG	153	21 (14)
	Flu-Bu-ATG (CGD/PMF)	13	1 (8)
	Flu-Bu-ATZ	4	0
	Flu-Cy	1	0
	Flu-sTBI MSD	2	0
	Ida-AraC	1	0
	RIC haplo	12	1 (8)
	RIC haplo ptCy	2	0

Bu, busulfan; Cy, cyclophosphamide; ATG, anti-thymocyte globulin; TBI, total body irradiation; ATZ, alemtuzumab; Flu, fludarabine; Mel, melphalan; VP16, etoposide; FLAMSA, fludarabine, Ara-C, amsacrine; CGD, chronic granulomatous disease; PMF, primary myelofibrosis; sTBI, single dose TBI (2 Gy); Ida, idarubicin; RIC haplo, reduced intensity conditioning for haploidentical HSCT; ptCY, post-transplant cyclophosphamide.
